# Breaking Bottlenecks for the TCR Therapy of Cancer

**DOI:** 10.3390/cells9092095

**Published:** 2020-09-14

**Authors:** Lena Gaissmaier, Mariam Elshiaty, Petros Christopoulos

**Affiliations:** 1 Department of Thoracic Oncology, Thoraxklinik at Heidelberg University Hospital, 69126 Heidelberg, Germany; lena.gaissmaier@med.uni-heidelberg.de (L.G.); mariam.elshiaty@med.uni-heidelberg.de (M.E.); 2 Translational Lung Research Center Heidelberg (TLRC-H), Member of the German Center for Lung Research (DZL), 69120 Heidelberg, Germany

**Keywords:** adoptive cell therapy, cancer immunotherapy, TCR therapy, gene editing

## Abstract

Immune checkpoint inhibitors have redefined the treatment of cancer, but their efficacy depends critically on the presence of sufficient tumor-specific lymphocytes, and cellular immunotherapies develop rapidly to fill this gap. The paucity of suitable extracellular and tumor-associated antigens in solid cancers necessitates the use of neoantigen-directed T-cell-receptor (TCR)-engineered cells, while prevention of tumor evasion requires combined targeting of multiple neoepitopes. These can be currently identified within 2 weeks by combining cutting-edge next-generation sequencing with bioinformatic pipelines and used to select tumor-reactive TCRs in a high-throughput manner for expeditious scalable non-viral gene editing of autologous or allogeneic lymphocytes. “Young” cells with a naive, memory stem or central memory phenotype can be additionally armored with “next-generation” features against exhaustion and the immunosuppressive tumor microenvironment, where they wander after reinfusion to attack heavily pretreated and hitherto hopeless neoplasms. Facilitated by major technological breakthroughs in critical manufacturing steps, based on a solid preclinical rationale, and backed by rapidly accumulating evidence, TCR therapies break one bottleneck after the other and hold the promise to become the next immuno-oncological revolution.

## 1. Immuno-Oncology’s Next Wave

The advent of immunotherapy was a crucial advance for medical oncology: for the first time ever, five-year survival became feasible for patients with highly lethal solid tumors, such as advanced melanoma and non-small-cell lung carcinoma (NSCLC) [1,2,3,4]. Immune checkpoint inhibitors (ICI), mainly PD-(L)1 blockers, are meanwhile approved for the treatment of most metastatic human cancers, while favorable evidence accumulates rapidly for earlier-stage diseases, as well [5]. Nevertheless, a significant fraction of patients does not benefit from contemporary immuno-oncologic (IO) options, the activity of which critically depends on adequate numbers of tumor-specific T-cells being present in the host and especially in the tumor microenvironment (TME) [6]. Hence, adoptive cellular therapies (ACT) are the currently rising next IO wave, aiming to fill this gap. They represent the most rapidly expanding sector of modern cancer immunotherapy and comprised 31% of the entire IO pipeline as of August 2019 [7].

## 2. The Unique Potential of T-Cell-Receptor (TCR) Therapy

Historically, the first source of tumor-specific T cells have been the patient’s own tumor-infiltrating lymphocytes (TIL), which can be isolated from tumor tissue, expanded using cytokines together with feeder cells or antibody-coated beads, and reinfused after lymphodepleting conditioning with subsequent IL-2 support [8]. Although this approach has consistently shown response rates of up to 50% in pretreated melanoma patients [9] and is technically feasible for lung and other solid cancers, as well [10], it has one major drawback: most epithelial cancers are poorly immunogenic, so that only a tiny fraction of harvested lymphocytes are actually active against the tumor, and reinfusion of bulk TIL does not result in responses [11]. In order to improve outcome, tumor-reactive T cells can be selected among bulk TIL based on the surface expression of various markers, such as PD-1 or CD137, and/or the tumor’s neoantigenic profile. However, these pipelines are still too time-consuming for routine application, with turn-over times of several weeks, and the product quality remains impaired by the T-cell exhaustion induced already before harvesting in the tumor microenvironment (TME) [12,13].

Genetic engineering of T cells obtained from the patients’ blood by leukapheresis overcomes both problems. These can be transduced with antigen receptors directed against tumor antigens, either classical T-cell (TCR) or chimeric (CAR), expanded and reinfused in a similar manner [14]. Although CAR-T cells are currently more advanced in clinical development and already approved for the treatment of various CD19^+^ hematologic malignancies [15], TCR-T cells combine several important advantages and are expected to become the mainstay of ACT for solid tumors [16,17,18].

In the first place, the number of antigens amenable to TCR-based therapies is much higher than of those for CAR, as less than 25% of human proteins are membrane-bound, and a considerably lower fraction of all amino acid sequences (probably <10%) will be accessible on the cell surface [17,19] (Table 1). While CARs can recognize only extracellular proteins, glycoproteins, glycolipids or carbohydrates through their single-chain variable fragment (scFv) [20], the peptide-major histocompatibility complex (pMHC) combinations recognized by the TCR are drawn from both the intra- and extra-cellular compartment [21]. Moreover, TCRs require lower amounts of antigen for activation than CARs, (generally 1–50/cell vs. >10^3^/cell), which is possibly linked to additional involvement of the CD4 or CD8 co-receptors [22], a higher number of immunoreceptor tyrosine-based activation motifs (10 vs. 3), and the ability of different TCR molecules to serially engage the antigens of low abundance, which amplifies responses [23,24,25,26]. At the same time, the lower target density and physiologic (for a T-cell) binding affinity (typically in the micromolar range for naturally occurring TCRs, compared to the nanomolar affinities of CARs [21]) facilitate a deeper penetration of TCR-T cells into solid tumors, while CAR-T cells can be halted at the outer tumor layer [17]. The downside is MHC-restriction, with most TCR-T trials focusing on HLA-A*0201-positive patients, who comprise approximately 50% of Caucasians [27].

When targeting solid tumors, the advantages of TCR over CAR are especially relevant and acquire critical importance. While several differentiation antigens expressed in the hematologic lineages are dispensable and therefore suitable for immunologic attack, for example CD19, CD33 and BCMA [28], there is no corresponding target that can be safely eliminated in epithelial cancers. TCRs directed against tumor-associated antigens (TAA) can result in serious off-tumor on-target toxicity, for example uveitis, labyrinthitis, vitiligo, and death of melanoma patients upon targeting the melanocytic differentiation antigens MART-1 and gp100, due to cross-reaction with normal melanocytes and cytokine release [29,30]. In addition, naturally occurring TCRs against TAA and cancer-testis antigens (CTA) are usually of low affinity, as TCRs with higher affinity against self-antigens are subject to negative selection in the thymus [31]. This is problematic, because lower TCR affinity has generally been linked to weaker T-cell responses in vitro and in vivo [32]. Immunization of transgenic humanized mice with human TAA to circumvent self-tolerance, amino acid substitutions, T-cell display systems and other methods are available to engineer higher-affinity TCRs [33,34,35,36,37], but also pose a significant safety risk, as illustrated by a clinical trial of a high-avidity TCR against the carcinoembryonic antigen (CEA): three patients nearly died of severe inflammatory colitis, because the transduced T cells attacked normal colonic epithelial cells, which also express CEA, albeit at lower levels [38]. In another trial using an HLA-A*0201-restricted, affinity-enhanced TCR targeting MAGE-A3/9, unexpected recognition of an HLA-A*0201-restricted MAGE-A12 epitope in the brain caused fatal neurotoxicity in two patients [39], while unexpected cross-reactivity of another affinity-enhanced TCR against HLA-A*01-restricted MAGE-A3 with the myocardial protein titin lead to cardiogenic shock and subsequent death of the first two treated patients [40]. Counterexamples of exceptional anti-tumor efficacy without off-target toxicities also exist [41,42], but testing for potential cross-reactivity is now routinely performed during the pre-clinical development of all novel TCRs [43]. 

Another critical problem for ACTs is the susceptibility of single-target approaches to tumor escape via antigen-loss. Clinical trials of CD19-directed CAR-T cells have indeed shown treatment failures to be mostly CD19-negative [44,45], and the same limitation also pertains to TCR-based ACT, necessitating the use of multivalent products [46]. Based on preclinical models, it has been proposed that a combination of TCRs targeting three or more mutant peptides with adequate affinity may be necessary and sufficient to eradicate established cancers [47]. Considering the scarcity, low efficiency, and poor safety of TAA and CTA, as outlined in the previous paragraph, exploitation of the much larger and much more tumor-specific reservoir of neoantigens becomes unavoidable for ACT. For the approximately 12% of human cancers attributable to oncogenic viruses, targeting oncoviral antigens would also offer similar advantages [48]. However, at the same time this also dictates the use of TCR-T over CAR-T cells, because neo- and onco-viral antigens are generally intracellular and can only be targeted by the former (Table 1) [17]. The essential tumorigenic role of many neoantigens (especially public ones, such as those generated by activating oncogene mutations) compared to TAA, that hinders antigen-loss as an evasion mechanism [21], the higher affinity of TCR directed against tumor-specific antigens (TSA) compared to TAA/CTA, and the better tumor penetration of TCR-T compared to CAR-T cells, are additional features of key importance for the treatment of solid tumors, but there are some critical bottlenecks to overcome first.

## 3. Critical Tasks

### 3.1. Neoantigen Multitargeting

First and foremost, both the development of multispecific cellular therapies and targeting of neoantigens currently pose serious technical challenges and have mainly been evolving along separate lines. For example Immatics (https://immatics.com/), a biotech company focusing on TCR-T therapy, has initiated a proof-of principle study investigating the safety and feasibility of a multitargeted ACT approach, focusing on multiple TAA, not neoantigens (NCT02876510) [49]. The patients’ tumors are biopsied and screened for expression of collagen VI alpha 3 exon 6 (COL6A3), PRAME, MAGEA1, MAGEA4, MAGEA4/8, NY-ESO-1, or matrix remodeling-associated protein 5 (MXRA5). After selection of up to four targets (if expressed by the tumor), T cells capable of recognizing these targets are isolated from the patient’s peripheral blood, activated, and expanded before reinfusion as a personalized multi-target TCR-T therapy. On the other hand, several TCR-T studies are targeting single public neoantigens, such as those arising from *KRAS* G12V restricted on HLA-A*1101 (NCT03190941) or hotspot *TP53* mutations [50]. Although these ACTs would be effective for several patients (i.e., all sharing the respective HLA-allele and harboring tumors with the respective neoantigen), their target population is nevertheless limited and their effectiveness is compromised by tumor-escape through antigen loss; therefore an individualized approach targeting multiple neoantigens appears to be much more reasonable in the long run [46,47].

One first bottleneck for clinical development of such mutatome-based TCR-T therapies is currently neoantigen identification. The first step is usually whole-exome sequencing (WES) of tumor and normal tissue in order to identify non-synonymous mutations [51], followed by RNA sequencing (RNA-seq) in order to characterize the expression of altered sequences [52]. Of note, it is now possible to perform WES on cell-free tumor DNA (ctDNA) or circulating tumor-cell (CTC) DNA, which is enriched for mutations shared between primary and metastatic sites [20]. Subsequently, potential neoantigens are assessed for their capacity to be processed by the proteasome and presented on the patient’s MHC, either by bioinformatic analysis, or by mass-spectrometry-based immunopeptidomics [52,53,54,55]. Multiple studies have found that only about 1–2% of non-synonymous mutations result in neoantigens that are recognized by T cells [56]. In silico prediction of MHC-I binding for potential neoepitopes is mainly based on neural network algorithms, e.g., NetMHC, which are less accurate for infrequent HLA-I alleles, HLA-II molecules, and potential targets resulting from special alterations, e.g., long insertions/deletions, gene fusions, splicing aberrations, epigenetic changes, and posttranslational modifications [51,54]. Alternatively, peptides presented on HLA molecules can be eluted and their amino acid sequence determined using liquid-chromatography-coupled tandem MS (LC-MS/MS), which reduces the number of false positives compared to bioinformatic pipelines, and can occasionally detect cryptic peptides overlooked by in silico methods [57]. Still, while highly specific, immunopeptidomic approaches suffer from low sensitivity, especially for peptides that are less abundant and more difficult to ionize and fragment, or when the quantity of available tumor material is limited [52]. 

**Figure 1:**
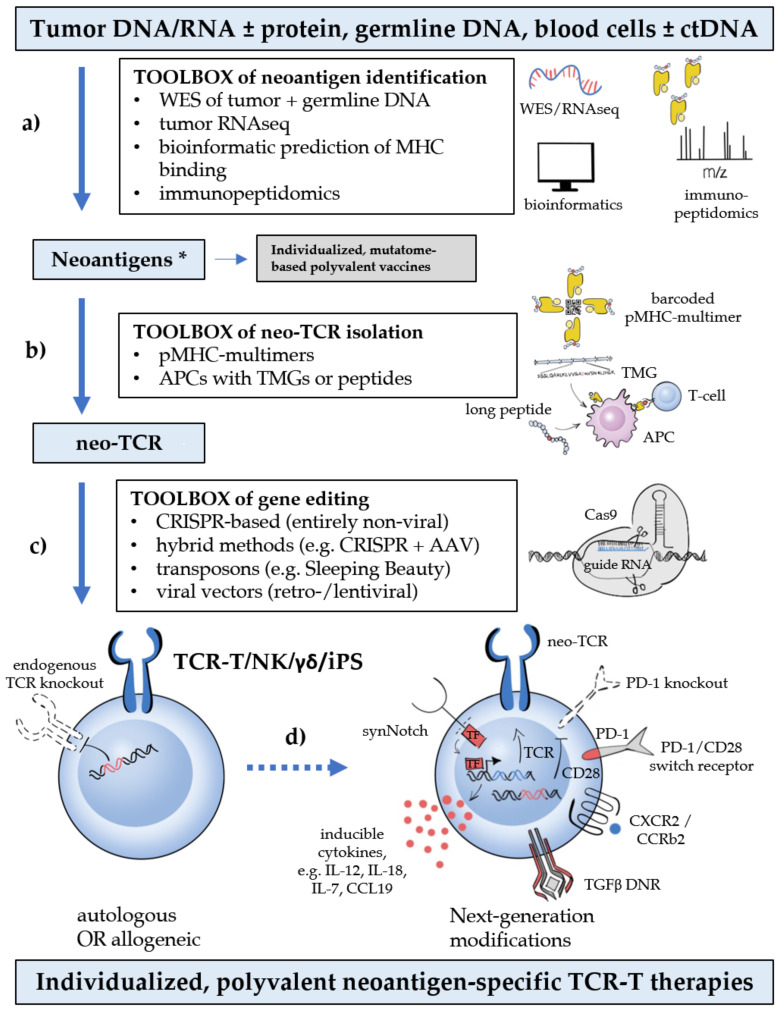
Critical steps, bottlenecks, and breakthroughs in neoantigen-based T-cell-receptor (TCR) therapy. Critical steps (blue boxes), bottlenecks (shown with lower-case letters: (**a**) rapid, high-throughput identification of public and private neoantigens; (**b**) isolation of neoepitope-specific TCRs (neo-TCRs); (**c**) (preferably non-viral) gene editing of autologous or allogeneic cells with concomitant knock-out of the endogenous TCR; (**d**) additional next-generation modifications to improve T-cell physiology), and technological breakthroughs (white boxes) that drive progress in the TCR therapy of cancer. The term “third-generation ACTs” has been coined for products combining these new technologies [18]. Polyvalency currently entails manufacturing multiple mono-specific TCR-T cells, which are then pooled together or sequentially infused to the patient. * in case of virally induced tumors, oncoviral antigens are also tumor-specific and can be exploited similarly to the tumor neoantigens.

The significant technical progress in neoepitope identification has been instrumental for two proof-of-principle studies testing mutatome-based vaccination in melanoma patients [58,59]. Using the aforementioned tools, individualized vaccines with multiple (generally 10–20) neoepitopes could be prepared for each patient in real time, which demonstrated the feasibility of neoantigen multitargeting within the clinical routine. Furthermore, their improved clinical results compared to earlier TAA-directed vaccination efforts, with long-term tumor control in the majority of patients, highlight the superiority of multivalent and TSA-based over single-antigen and TAA-based strategies, and have paved the way for similar vaccination efforts in head-and-neck, bladder, lung and other cancers [47,60]. Notwithstanding, extension of the same principle to ACTs is dependent on two crucial additional steps: isolation of the respective neoepitope-specific TCRs, and their transfer into recipient cells using scalable methods in a timely manner (Figure 1) [61].

Identification of neoantigen-specific TCRs is achieved by testing the immunogenicity of potential neoepitopes against T cells [55]. Usually, these cells are collected from tumor biopsies or the patient’s blood, and can be enriched for tumor-reactive clones by sorting for CD137, CD39 and PD-1 positivity, in order to increase yield [62,63,64,65]. These can then be tested against large numbers of putative neoepitopes by high-throughput assays using barcoded pMHC-peptide multimers in microfluidic systems with a high sensitivity (detection of down to 1 in 10^6^ neoepitope-specific cells), followed by isolation, profiling and TCR sequencing [62,66]. Suitable peptide-MHC-I multimers are meanwhile available for almost all patients (>95%), but interrogation of MHC-II remains problematic [52]. A more time-consuming T-cell screening method utilizes coculture with antigen-presenting cells (APCs) that are either transfected with “tandem minigenes” (TMGs) or pulsed with long peptides to present the patient’s neoepitopes [14,67,68]. A minigene consists of one non-synonymous mutation flanked by 12 amino acids of the wild-type sequence, and is merged together with 5–23 other similar sequences in tandem, followed by electroporation into autologous APCs, typically B cells or dendritic cells [52]. Alternatively, 25-amino-acid-long peptides encompassing the mutated amino acid can be synthesized and pulsed onto the APCs. In both cases, APCs are cocultured with either TILs or peripheral blood T cells, the reactivity of which can be assessed by ELISPOT or the upregulation of T-cell activation markers (e.g., CD137, CD134) [69,70,71]. APC-based screening is less biased than approaches relying on pMHC-multimers, and has a special advantage in the identification of neoantigen reactive CD4^+^ T cells. Nevertheless, costs for both methods are still high, and availability of APCs or T cells can be limiting, especially for tumors with a high tumor mutational burden (TMB) and a large number of candidate neoepitopes [52]. One solution to is to perform the screening using allogeneic T cells and APCs from healthy donors (HLA-matched or partially matched), which could recognize human tumors from different hosts in several studies, including neoantigens ignored by the patient’s own T cells [72,73,74,75]. An additional advantage of the allogeneic approach is considerable shortening of the procedure, which is essential for patients with metastatic malignancies [76]. Modern protocols can identify neoepitope-specific T cells from healthy donor T-cell repertoires in only 2 weeks [73,77,78]. On the downside, using allogeneic TCRs requires thorough evaluation for self-reactivity, as these have not been subject to selection by the patient’s thymus [47]. Currently, the fastest pipelines can complete all steps of neoantigen-specific T-cell isolation, from mutation calling to validation of immunogenicity, in 6 weeks [62].

Finally, the identified neoepitope-specific TCRs are transferred into suitable recipient cells, which are subsequently expanded to form the cellular therapeutic. Retro- and lentiviral vectors have been the mainstay for T-cell engineering for years and are still widely used. However, these procedures are associated with significant biosafety hazards that limit availability, are very expensive (approximately a quarter million USD per gene transfer), time-consuming (several weeks), and not suitable for the upscaling necessary to meet the increasing demand imposed by neoantigen multitargeting in conjunction with other modifications (Figure 1) [18,21]. Therefore, non-viral methods of targeted integration into the *TCR* locus have emerged as the preferred alternative, and offer the additional advantage of concomitantly disrupting the endogenous TCR, which prevents graft-versus-host disease, TCR mispairing and competition for signaling components [18]. Most promising is an entirely non-viral, CRISPR-based approach, which allows efficient, site-specific insertion of large DNA sequences (>1 kb) in the genomes of primary human T cells within 1 week, while preserving cell viability [79]. Other methods combine non-viral TCR disruption (e.g., via CRISPR, zinc-finger (ZFN) or transcription activator-like effector nucleases (TALENs)) with transduction [80,81,82,83,84], or utilize electroporation of transposons, which also reduces manufacturing costs and duration compared to viral vectors, but may impair cell viability [85] and result in a lower transgene expression [86]. Pivotal studies of CRISPR editing with ssDNA as donor template for homology-directed repair have shown a very low (0.01%) off-target integration rate [79], but genetic characterization of gene-edited cells remains essential to ensure safety [18]. Further quality measures include validation of the neoepitope specificity for TCR engineered cells and screening for off-target reactivity. Clinical trials of non-viral gene editing to simultaneously target multiple neoantigens in various cancers are currently underway (e.g., NCT04102436 and NCT03970382, Supplementary Materials Table S1).

Deployment of several different neoepitope-specific TCRs for targeting multiple neoantigens in each patient classically entails manufacturing multiple mono-specific TCR-T cells, which are then pooled together or sequentially infused to the patient. However, experience with CAR-T cells has shown that co-expression of two different antigen receptors on the same T-cell results in a higher potency than pooling two different monospecific T-cell populations together [87]. The presence of naturally occurring dual-TCR T cells in the human immune system (estimated as about 10% of αβ T cells) and their enhanced alloreactivity [88,89] actually suggest that bispecific TCR-T cells might be a viable ACT option, but this remains to be explored. 

Current ACTs mainly depend on CD8^+^ T lymphocytes as cytotoxic executors [90]. However, ample evidence suggests that concomitant mobilization of CD4^+^ T cells against the tumor is essential for epitope spreading and the durability of CD8^+^ T-cell responses [91,92]. Serious obstacles to such combined strategies at present are the low efficiency of technologies for MHC-II neoepitope identification and pMHC-II-specific TCR isolation, as already outlined. In addition, most solid tumors express only pMHC-I, which natural CD4^+^ cells cannot recognize, while equipping them with pMHC-I-specific TCRs does not entirely solve the problem, because these generally need the participation of a CD8 co-receptor to engage the antigen [17]. One emerging solution is co-transduction with CD8α homodimers or CD8αβ, which can significantly enhance CD4^+^ TCR-T-cell activation and cytokine production in preclinical models [93,94]. Besides, many neoantigen-specific TCRs have a high pMHC affinity and could therefore elicit T-cell activation regardless of CD8 [95].

Beyond normal T lymphocytes, several other cell types can also be equipped with a TCR for neoantigen targeting and offer special advantages. TCR-transduced NK cells retain their natural cytotoxicity in addition to the newly-acquired MHC-restricted capabilities, and are therefore resistant to HLA-loss, which is a main mechanism of tumor evasion under TCR therapy [96,97,98]. Induced pluripotent stem cells (iPS) can be used to transform derived lymphocytes or other mesenchymal cells to cytotoxic T cells of any specificity with extended proliferative potential [99]. Gamma-delta T cells transduced with tumor-specific αβTCRs acquire MHC-restricted cytotoxic potential without the problem of TCR mispairing [100]. Of note, all aforementioned parental cell types have reduced or no alloreactivity and could also form the basis for readily available “off-the-shelf” ACTs [96,100,101].

### 3.2. Next-Generation Products

Optimization of TCR specificity, while representing the first and crucial step, is far from the finish line in the development of an engineered T-cell therapeutic solution. Countless other interactions before and after the TCR-pMHC engagement are critical for T-cell function and need to be taken into consideration, as well. In fact, the single most important step for the breakthrough of CAR-T cells was not related to their antigen-specificity, but rather came through other improvements. Early, first-generation CARs contained only the intracellular part of the CD3ζ chain and showed little capacity to initiate an immune response in transgenic mice due to insufficient CAR-T cell activation and proliferation upon antigen engagement [102]. Only after supplementation with additional co-stimulatory receptor domains, CD28 or 4^-^1BB-derived [103,104], could these second-generation CARs deliver the efficacy that ultimately led to CAR-T cell approval for hematological malignancies by the U.S. Food and Drug Administration (FDA) in 2017 [105]. Improved understanding of T-cell physiology and the additional challenges posed by the microenvironment of solid tumors continuously shape further “next-generation” improvements. Many of them were first implemented in CAR-T cells, which are more advanced in development, but all are equally relevant for TCR therapies, as well. Indeed, several have already been successfully introduced into the TCR-T space, while many others are in the process of being transferred. Broadly, they can be categorized as follows.

#### 3.2.1. T-Cell Persistence, Memory, and Fitness

Experience from CAR-T trials shows that T-cell persistence in the host is a critical factor for durable clinical benefit [106]. At the same time, accumulating evidence suggests that engineered T cells of the naive and particularly the central memory subsets can survive longer in vivo as memory cells after transfer [107,108]. Purification of these T-cell subsets for use in ACT can be achieved either with fluorescence-activated cell sorting, meanwhile also available in closed systems, or with immunomagnetic isolation [109]. In both procedures, the labeling method requires special attention, because the employed molecules remain bound to the cells and could pose a health risk to recipients, except for Fab-streptamers, which can be removed by subsequent treatment with d-biotin [110]. Furthermore, the fact that many ACT candidates are heavily pretreated advanced cancer patients with impaired T-cell function, has sparked interest for alternative ACT sources, i.e., other cell types or T cells from unrelated healthy donors, with better fitness and broader availability [111]. Manufacturing using homeostatic cytokines, like IL-7 and IL-15, instead of the previously standard IL-2, contributes to preservation of earlier differentiation, i.e., memory stem and central memory phenotypes, that mediate superior antitumor effects [112,113]. Genetic engineering to overexpress c-Jun, a protein that combines with c-Fos to form the transcription factor AP-1, can also increase expansion and prolong survival of adoptively transferred T cells after reinfusion by driving transcription of IL-2 [114].

Nevertheless, functional deterioration of effector T cells remains a major problem, mainly caused through chronic activation by the tumor. The ensuing “T-cell exhaustion” (Tex) is characterized by a hierarchical loss of effector functions (e.g., cytokine production, proliferation and killing), altered cell metabolism, aberrant transcriptomic and epigenetic profiles, as well as increased expression of multiple inhibitory receptors (IR), such as Cytotoxic T-Lymphocyte-Associated Protein 4 (CTLA-4), Programmed cell Death protein-1 (PD-1), T-cell immunoreceptor with IG and ITim domains (TIGIT), Lymphocyte-Activated Gene-3 (LAG3), 2B4, B-and-T-Lymphocyte-Attenuator (BTLA), and T-cell Immunoglobulin and Mucin-containing protein 3 (TIM3) [115,116]. In a vicious cycle, these IR aggravate the Tex phenotype by providing signals that further attenuate T-cell function and have therefore become targets of genetic ACT engineering. One strategy is to knock them out using multiplexed CRISPR-based gene editing [80] or other non-viral methods: e.g., the inactivation of *PDCD1* in melanoma-reactive CD8^+^ T cells and fibrosarcoma-reactive polyclonal T cells using TALEN-enhanced T-cell infiltration in the tumor site and tumor control [117]. Alternatively, the function of IR can be neutralized, for example by engineered PD-1 “dominant negative” receptors (DNR) lacking signaling motifs, or even reversed by chimeric PD1/CD28, CTLA-4/CD28, TIGIT/CD28 and CD200/CD28 “switch” receptors that fuse IR exodomains with costimulatory endodomains to increase cytokine secretion and anti-tumor activity [118,119,120,121,122,123]. The same concept can be applied to immunosuppressive cytokines, for example tumor derived IL-4 and TGFβ that promote tumor growth and attenuate T-cell activity in several tumor types [124,125]: fusing the exodomain of the IL-4 receptor with the endodomain of the IL-7 receptor sustained the Th1 immune phenotype of transferred T cells and prolonged the survival of recipient mice in a xenograft model [126], while T cells transduced with both a tumor-specific TCR and a TGF-β DNR resulted in an improved tumor treatment at various dose levels compared with control T cells in transgenic mice [127]. Co-expression of a TGFβ DNR is currently being examined in a basket phase 1/2 clinical trial (NCT02650986) of NY-ESO-1 specific TCR-T cells by the Roswell Park Cancer Institute in collaboration with the National Cancer Institute (NCI) [128]. Providing T cells with additional activating receptors appears to produce similar effects, e.g., transfer of the 4-1BB gene along with F4-TCR (a MART-1 specific TCR) resulted in upregulation of activation markers, enhanced cytokine production, and a relative resistance to the immunosuppressive effects of TGFβ [129]. Finally, efforts to modify the TCR itself have also yielded promising results, for example, Miyao et al. generated artificial T cell-activating adapter molecules (ATAM) by inserting the intracellular domain of either CD28 or 4-1BB into the ζ-chain of CD3 and observed superior proliferation and antitumor effects both in vitro and in a murine model [130]. However, a major limitation of current approaches to counter T-cell exhaustion is the epigenetic stability of Tex, which limits durability of reinvigoration [131]. Therefore, much hope is set on epigenetic reprogramming of T cells via disruption of genes controlling DNA methylation, which could complement and potentiate other strategies against T-cell exhaustion in the future [132].

#### 3.2.2. Navigating and Prevailing in the TME

In contrast to many hematological malignancies, ACT for solid tumors are massively challenged by the additional need to penetrate and endure an adverse TME. The process of T-cell migration to the tumor site is led by chemotactic gradients [133], which has prompted transduction of chemokine receptors, such as CXCR2 and CCR2b, in genetically modified T cells to improve trafficking (Figure 1) [134,135,136,137]. Furthermore, additional transfer of inducible cytokine expression cassettes, for example for IL-12, IL-18, IL-7, CCL19, can augment tumor regression by increasing infiltration and activation of immune cells in the TME (Figure 1) [138,139,140]. “Heating-up” the TME is especially important in cases of immune-altered or immune-desert tumors, in which the multiple immunosuppressive mechanisms (hypoxia, nutrient-deprivation, inhibitory immune and mesenchymal cells, e.g., regulatory T cells, myeloid-derived suppressor cells, cytokines, e.g., IL-10) cannot be effectively overcome by ACT alone [141,142,143]. Any mediator of innate immunity can be potentially incorporated into “armored” TCR-T cells or given concomitantly with them in order to overcome the resistance of “cold” tumors [144]. For example, transfer of Fms-like tyrosine kinase 3 ligand (FLT3L), a dendritic cell (DC) growth factor, to T cells increased DC recruitment, T-cell activation and epitope spreading [145], while additional administration of agonistic anti-4-1BB and polyinosinic:polycytidylic acid (poly I:C), a Toll-like receptor 3 agonist, further enhanced immune responses [145]. Another example is implantable biopolymer devices to co-deliver engineered T cells and STING agonists directly to the surfaces of solid tumors: the resulting prolonged exposure to both agents facilitated eradication of various tumors in orthotopic mouse models, including the notoriously immune-resistant pancreatic adenocarcinoma, as well as tumor cells not recognized by the adoptively transferred lymphocytes alone [146]. Similar data are also accumulated for several other immunotherapeutic strategies: VEGF inhibition in the form of VEGFR-2-specific CARs or anti-VEGF antibodies synergizes with ACT to increase T-cell recruitment, tumor regression and survival in murine melanoma models [147,148]; PD-(L)1 inhibitors counter tumor-induced T-cell attenuation in the TME, have demonstrated synergy with CAR-T cells and are already combined with TCR-T cells in clinical trials (e.g., NCT03709706) [149]; indoleamine 2,3-dioxygenase (IDO) inhibitors block a key enzyme of tumor-associated macrophages and have improved ACT efficacy in preclinical models [150]; radiotherapy [151], oncolytics [152], and several other immunomodulators [144]. Hence, combination of TCR-T with various other immunotherapeutics provides a practical solution to potentiate efficacy, until the working principle of the latter can be incorporated into a “next-generation” ACT via genetic engineering (Figure 1). Of note, cancer-associated systemic immune dysregulation can take very different forms according to the type of tumor, for example intense systemic immune activation with a B-cell-induced TCR signaling defect in case of indolent B-cell lymphomas [153,154], accumulation of hyporesponsive CD247-deficient naive T-cells with cortical thymomas [155,156,157], and varying immune reactivity in other epithelial tumors [158,159], which is an additional layer of potential individualization for ACTs, beyond the adjustment of antigen receptor specificities according to each patient’s profile of tumor neoepitopes. Hence, precise characterization of the immune abnormalities induced by the tumor could assist selection of the most suitable immunotherapeutic partners or “next-generation” modifications in each patient, in order to further “fine-tune” and personalize TCR-T and other ACT therapies in the future.

#### 3.2.3. Avoiding Toxicity

Besides enhanced efficacy, improved tolerability is also essential for wide-scale application of ACT. An inherent issue with TCR engineering is the coexistence of two different active TCR genes in the manipulated T cells allowing for mixed dimerization with unpredictable specificity, which can cause lethal graft-versus-host-disease (GvHD) in murine models [160,161]. Therefore, measures to prevent TCR mispairing, like murinization of constant TCR chains [162], codon optimization, cysteineization [163], or disruption of the endogenous TCR genes is imperative. In the past, the latter has necessitated additional manipulation with e.g., ZFNs, TALENs [83,84], or RNA interference (RNAi) [161,164,165], but can now be accomplished concomitantly with the TCR transfer, using newer, TCR locus-specific CRISPR-based gene editing methods [79,166,167]. The resulting T cells show physiological regulation and higher expression levels of the introduced TCRs [161,167,168], superior antitumor activity in vivo, and reduced GvHD mortality [83,161,164,166].

Additional measures tackle “on-target off-tumor toxicity”, for example, the use of inhibitory CARs (iCARs) in combination with the main antigen receptor directed against the tumor. This separate iCAR recognizes antigens expressed in normal tissues, but not tumor cells, and is fused to the signaling endodomain of an IR, e.g., PD-1, that prevents TCR-T autoreactivity [169]. A similar purpose is served by combinatorial antigen-sensing circuits, in which the recognition of one antigen, e.g., by a synNotch receptor, induces expression of an effector TCR or CAR directed against a second antigen, thus limiting the activity of ACT to cells expressing both targets (Figure 1) [170]. Suicide gene technologies, on the other hand, have been developed to treat ongoing adverse reactions. There is a wide variety of such technologies [171], most prominent among which are genes for the herpes-simplex-virus thymidine-kinase (HSV-TK) and the inducible Caspase9 system [172,173]. In the former, administered nucleoside analogs, such as acyclovir or ganciclovir, are phosphorylated by the HSV-TK and incorporated in the DNA, leading to chain termination and cell death [171,172]. Caspase9-based methods, on the other hand, rely on expression of a chimeric protein that dimerizes upon administration of a small synthetic molecule, and activates the apoptotic pathway that induces cell death [171,173].

## 4. Status of Clinical Development

**Figure 2:**
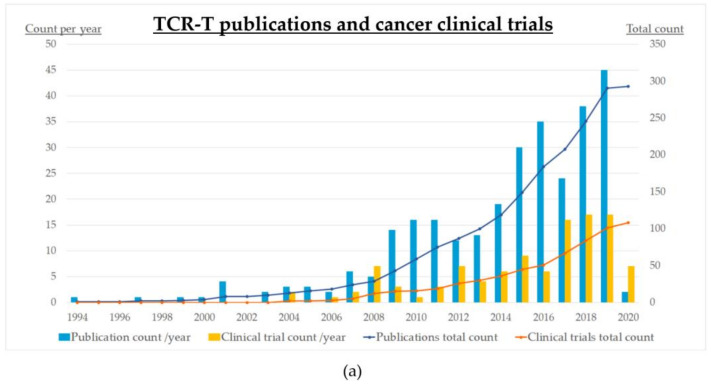

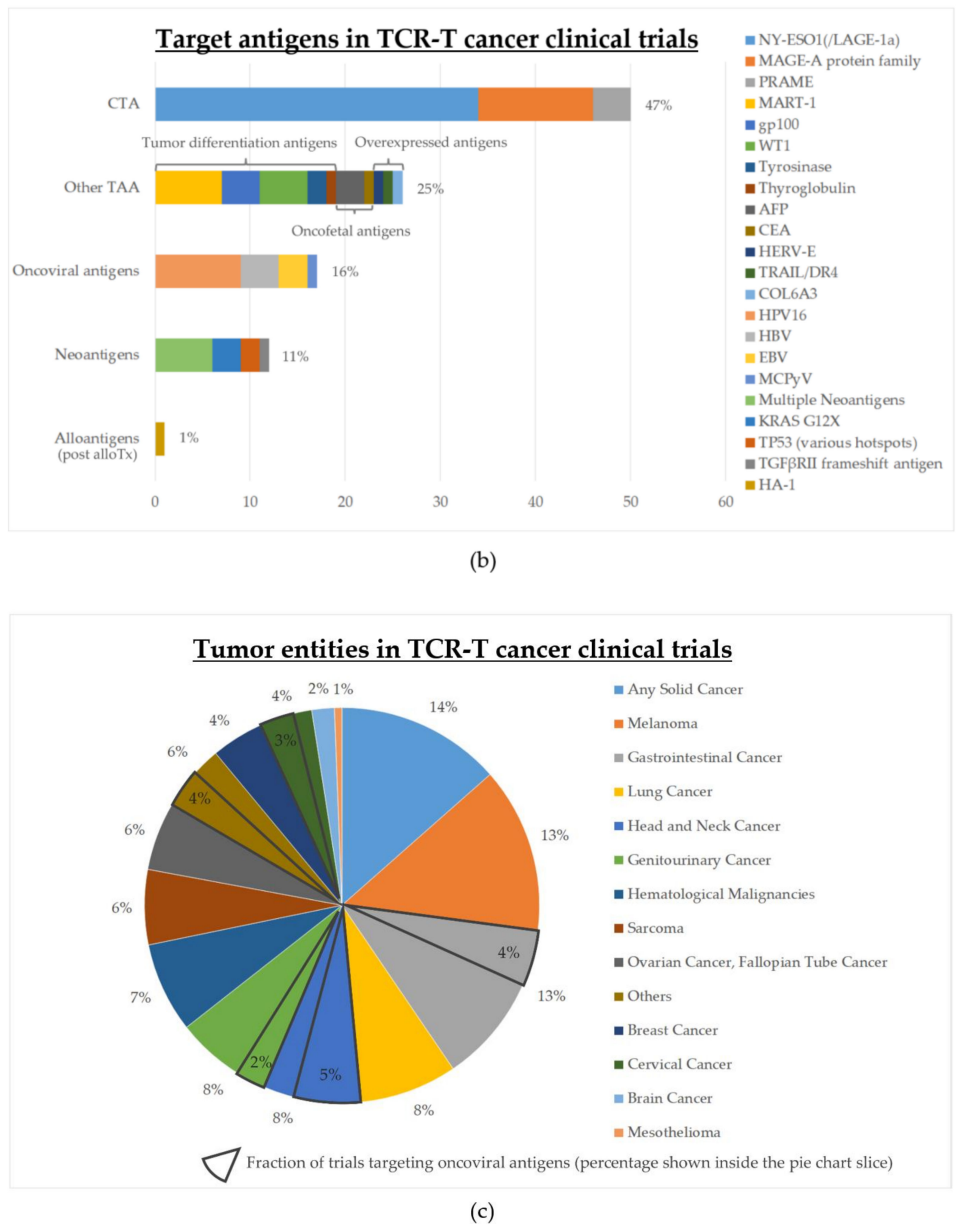
Status quo of clinical development for cancer TCR-T therapies as of June 2020: (**a**) numbers of clinical trials (*n* = 104) and publications (*n* = 293); (**b**) target antigens in the various clinical trials; (**c**) cancer entities in the various clinical trials; “others” includes vulvar (*n* = 3) and vaginal (*n* = 2) neoplasms, primary peritoneal carcinoma (*n* = 2), thyroid cancer (*n* = 1), and Merkel-cell carcinoma (*n* = 1). Clinical trials were identified by a search in ClinicalTrials.gov on 15 June 2020 using the keyword “TCR”, followed by filtering the results to include interventional trials for oncological entities only, and manually verifying which trials specifically employ genetically engineered TCR-T therapies (*n* = 104). Publications were identified by a search in PubMed using ((“Immunotherapy, Adoptive”[Mesh]) AND (TCR[Title/Abstract])) OR ((“Immunotherapy, Adoptive”[Mesh]) AND (T cell receptor[Title/Abstract])), which returned 853 entries, followed by manual verification of TCR-T therapies as the main subject (*n* = 293, publications on other ACT, e.g., CAR-T, and studies not involving TCR engineering, e.g., using transgenic mouse models, were excluded); alloTx: allogeneic hematopoietic cell transplantation.

The growing interest for cancer TCR-T therapies and the increasing number of promising strategies are reflected in the upsurge of publications and clinical trials during recent years (Figure 2a). The first oncological studies with engineered TCR-T cells were registered at clinicaltrials.gov as early as 2004, and with some fluctuations, their numbers have increased over the last 15 years leading up to a total of 104, including 20 withdrawn or terminated, and 17 completed protocols (Figure 2, Table 2 and Table S1).

All registered studies are either phase I or II, and the vast majority concern solid tumors rather than hematological malignancies (Figure 2b and Table S1). The most prevalent entity is melanoma (13%), followed by gastrointestinal cancers (13%), lung cancer (8%) and almost all other solid tumors (Figure 2b). Most frequent targets are CTA (47%) and other TAA (25%), while oncoviral antigens make up 16% (HPV in nine trials, HBV in four, EBV in three, MCV in one), and neoantigens make up 11% of trials (single in six, multiple individualized in six, Figure 2c). Next-generation TCR-T products are the subject of 8/104 trials (8%, involving PD-1, CD200, TGFβ, IL-12, CD8α, and the Caspase9 switch, details are given in the Table S1), while only 6/104 trials (6%) pursue individualized targeting of multiple neoantigens (Table S1) with the earliest one registered in 2018 (Table 2). Of these, two are performed at the NCI under the direction of Dr. S. Rosenberg (NCT03412877 using viral gene editing, NCT04102436 using the sleeping beauty transposon system in cooperation with Ziopharm, and NCT04194190 as expanded access to neoantigen-based polyvalent TCR gene therapy for a single breast cancer patient, without further technical details); one is performed by PACT Pharma in California, USA using its proprietary platforms for high-throughput neoantigen-specific TCR isolation and non-viral gene editing (NCT03970382); and two are performed in Guangzhou, China (NCT03778814 by the Second Affiliated Hospital of Guangzhou Medical University, and NCT03891706 by FineImmune Biotechnology Co., LTD, without further technical details). Non-viral gene editing as implemented within the NCT04102436 trial is the newest development to enter clinical testing, with an estimated start date on 24 August 2020.

## 5. Conclusions and Perspectives

The field of oncological TCR therapies is extremely versatile and evolves rapidly. Currently, two elements emerge as most promising for therapeutic potential and future clinical impact: individualized, mutatome-based strategies utilizing high-throughput neoantigen-specific TCR-isolation and expeditious non-viral gene editing; as well as “next-generation” modifications that improve T-cell physiology and boost immune activation in the adverse solid tumor microenvironment. Facilitated by major technological breakthroughs, both can meanwhile be realized within clinical trials and herald the next immuno-oncological revolution.

## Figures and Tables

**Table 1:** Comparison of chimeric antigen (CAR) and T-cell receptors (TCRs).

	CAR	TCR
Target Ag	Surface proteins, glycoproteins, glycolipids, carbohydrates	Peptides from surface and intracellular proteins
Ag recognition	MHC-independent	MHC-dependent
Receptor structure	Single-chain, scFv 3 ITAMs	αβ heterodimer 10 ITAMs
Affinity for target	Nanomolar range	Micromolar range
Required target density for response	>10^3^/cell	∼1–50/cell

Ag: antigen.

**Table 2:** TCR-T trials against cancer-specific or cancer-associated antigens in clinicaltrials.gov.

Type of Antigen	Number of Trials	% of Active Trials ^1^	% of Completed Trials	Start Year of the First Trial
Cancer-testis antigens (CTA)	50	56% (28/50)	12% (6/50)	2008
Other tumor-associated antigens (TAA)	25	44% (11/25)	28% (7/25)	2004
Oncoviral antigens	17	88% (15/17)	12% (2/17)	2014
Neoantigens (public or private)	12	75% (9/12)	8% (1/12)	2006 (public) 2018 (private)

The entire dataset is given in the Table S1; ^1^ active: “recruiting”, “not recruiting” and “not yet recruiting” trials.

## Supplementary Materials

The following are available online at https://www.mdpi.com/2073-4409/9/9/2095/s1, Table S1: TCR-T cancer clinical trials.
